# Impact of religious fasting on ocular dryness: objective and subjective assessment

**DOI:** 10.3389/fmed.2025.1488765

**Published:** 2025-02-05

**Authors:** Amal F. Alomari, Sara Issa, Asma Musleh, Mohammad Abusamak, Omar Bdair, Saif Aldeen AlRyalat, Alanoud Al-Wakfi, Mohammed Jaber, Ahmad Alloubani, Haitham Sahawneh, Muhannd El-Faouri, Ayman Abdul Aziz, Muawyah Al Bdour

**Affiliations:** ^1^Department of Special Surgery, Al-Balqa Applied University, Al-Salt, Jordan; ^2^Department of Ophthalmology, Amman Eye Clinic, Amman, Jordan; ^3^Faculty of Engineering Technology, Al-Balqa Applied University, Amman, Jordan; ^4^Faculty of Medicine, Memorial University of Newfoundland, St. John’s, NL, Canada; ^5^Department of Ophthalmology, School of Medicine, The University of Jordan, Amman, Jordan; ^6^Department of Ophthalmology, Houston Methodist Hospital, Houston, TX, United States; ^7^National Center for Diabetes, Endocrinology and Genetics, Amman, Jordan; ^8^Department of Ophthalmology and Visual Sciences, Case Western Reserve University, Cleveland, OH, United States; ^9^University Hospitals Eye Institute, University Hospitals Cleveland Medical Center, Cleveland, OH, United States; ^10^Department of Special Surgery, Faculty of Medicine, Hashemite University, Zarqa, Jordan

**Keywords:** fasting, lubrication, Ramadan, tear, OSDI questionnaire, dry eye

## Abstract

**Background:**

Certain religions require long hours of fasting, abstaining from fluid intake for durations extending up to 16 h. Lack of fluid intake may alter multiple physiological parameters, which can influence the ocular system. In this prospective study, we evaluated the effect of prolonged fasting on dry eye disease using both objective and subjective measures.

**Methods:**

We included patients who fasted for at least 12 h a day for at least 2 weeks, including the testing day, and retested them at least 1 week after the fasting period had ended with no fasting on the testing day. At each visit, Non-Invasive Keratograph Break up time (NIKBUT) and Tear meniscus height (TM) were measured using the Oculus Keratograph 5 M. Ocular Surface Disease Index (OSDI) was evaluated at each timepoint to assess dryness symptoms subjectively.

**Results:**

This study included a total of 40 patients. NIKBUT values during the fasting times were higher than during the non-fasting times; however, the difference was statistically non-significant. There were no significant differences in TM and OSDI measurements between non-fasting and fasting periods (*p* > 0.05). Lubricating eyedrop use was significantly lower in fasting patients.

**Conclusion:**

Our study showed that prolonged fasting, including complete abstinence from fluid intake, did not lead to significant dryness, neither subjectively nor objectively. During fasting, patients used significantly fewer lubricating drops compared to non-fasting periods.

## Introduction

Fasting during the holy month of Ramadan, a fundamental practice within Islam constitutes one of the five pillars of the faith. This religious observance involves refraining from eating and drinking between dawn and sunset throughout the entire month. Despite its numerous acknowledged benefits for the human body (1), Ramadan fasting induces several physiological changes in the ocular system (2). These alterations encompass variations in tear film protein, osmolarity, and secretion (2, 3), potentially arising from significant shifts in sleeping patterns and dietary habits, accompanied by intermittent dehydration during fasting periods (4, 5). While numerous studies have explored the impact of fasting on ocular health (2, 3, 5–10), the majority of these investigations employed slit lamp examinations to evaluate dry eye syndrome.

In our research, we opted for a more objective approach, utilizing the OCULUS Keratograph^®^ 5 M to assess parameters associated with tear film secretion and stability. This method surpasses conventional techniques in dry eye assessment due to its enhanced objectivity. The primary objective of our study was to evaluate the influence of prolonged fasting on eye dryness, employing both objective measures with the OCULUS Keratograph^®^ and subjective assessments using the Ocular Surface Disease Index (OSDI).

While numerous studies have explored the impact of fasting on ocular health (2, 3, 5–10), the majority of these investigations employed slit lamp examinations to evaluate dry eye syndrome.

## Materials and methods

This is a cohort prospective study that was conducted at two timepoints and was carried out from April to August 2023. The first fasting timepoint was in the last 2 weeks of Ramadan between 6th of April to 20th of April. The second non-fasting timepoint involved bringing back the same patients after Ramadan. To address the uncertainty of the time between fasting and non-fasting periods, we ensured that all participants met strict inclusion criteria, with fasting durations of at least 12 h per day for a minimum of 2 weeks. Additionally, non-fasting data were collected after a standardized period of at least 1 week following the end of fasting, ensuring consistency in the study design. This study was conducted at the National Center for Diabetes, Endocrinology and Genetics. All procedures performed were after approval of the National Center for Diabetes, Endocrinology and Genetics review board and were conducted in concordance with the latest Helsinki Declaration. All patients signed an informed consent at the beginning of the study.

### Participants

We included patients who had at least 12 h of fasting, including no food or water intake. Recruiting patients achieved this during the month of Ramadan, which is the ninth month in the Islamic calendar. During Ramadan, Muslims abstain from eating and drinking from dawn until sunset (12–17 h daily for 1 month). We included patients with the following criteria:

- No previous corneal refractive surgery or ocular surface disease, where a thorough history and complete slit lamp exam were performed.
- Fasting (complete abstinence from food and water) between dawn to sunset for at least the past 2 weeks, including the day of the exam.
- Complete abstinence from food and water for at least 12 h on the day of exam.

Exclusion criteria included patients having history of collagen/vascular tissue disease, patients taking topical antiglaucoma medications, patients with ocular surface diseases including blepharitis, trachoma and allergic conjunctivitis; patients with a history of previous refractive surgery; and patients who are known to wear contact lenses. Each included patient was informed that a follow up exam will be performed at least 1 month after initial exam, after the month of Ramadan ends.

For the follow up visit, we adopted the following inclusion criteria:

- Completed the initial baseline exam during the month of Ramadan.
- No fasting for the past week, including the day of the examination.

### Variables

The same clinical tests were performed at each timepoint. Each patient was tested using Oculus Keratograph. The Keratograph 5 M is an advanced corneal topographer with a built-in real keratometer and a color camera that uses white or infrared illumination to determine the break up time using the Non-Invasive Keratograph^®^ Break-Up Time (NIKBUT) procedure. The (NIKBUT) was recorded as the number of seconds that elapse between the last blink and the appearance of the first dry spot in the tear film using infrared illumination. What is unique about this keratograph is that it uses infrared light which is invisible to the human eye and produces no glare, hence no reflex tearing is caused by this examination. The Keratograph^®^ 5 M has been evaluated and shown to provide acceptable repeatability and reproducibility in previous studies (11, 12). The tear meniscus height was precisely measured using an integrated ruler in mm (TM).

Subjective symptoms were measured using an Arabic translation of the Ocular Surface Disease Index (OSDI) survey. This is a self-administered 12-item questionnaire that provides a global measure of dry eye. The OSDI score ranges from 0 to 100, where a score of 0 indicates no disability and 100 indicates extreme disability. Comparison of the results of the OSDI questionnaire, NIKBUT, and TM at the two timepoints was performed and used as the standard indicators of dry eye disease in subjects during and after Ramadan fasting.

### Sample size calculation

We used G-power (Version 3.1.9.3) for sample size calculation. We determined an effect size of 0.55, where we calculated the effect size using a previous study that studied a dryness related parameter (difference in tear secretion) between fasting and non-fasting (2). With an alpha level of 0.05, a power of 90%, we calculated a total sample size required to be 37.

### Statistical analysis

We used IBM SPSS Statistics for Windows, version 26.0 (IBM Corp., Armonk, N.Y., United States) in our analysis. We used mean (± standard deviation) to describe continuous variables. We used count (frequency) to describe other nominal variables. We calculated the mean difference during fasting minus non-fasting states with standard deviation and compared the mean difference using paired sample t test. We adopted a *p* value level of 0.05 for significance threshold.

## Results

The mean age of the patients was 38.17 years with standard deviation of 16.03. They were 13 men (32.5%) with a mean age of 38.38 ± 17.78 and 27 women (67.5%) with a mean age of 38.07 ± 15.48, with no significant age difference (*p* = 0.487). Eight (20%) patients were diabetics. Majority of patients did not use lubricating drops within a few weeks, including 34 (85%) during fasting and 32 (80%) during the non-fasting periods (Table 1).

**Table 1:** Descriptive statistics of included samples during fasting and non-fasting periods.

		Mean	Std. Deviation	Count	%
Age		38	16		
Gender	Male			13	32.5
	Female			27	67.5
Non-fasting last use of lubricating eye drops	Few hours ago			7	17.5
	Few days ago			1	2.5
	More than a week ago			32	80
Fasting last use of lubricating eye drops	Few hours ago			1	2.5
	Few days ago			5	12.5
	More than a week ago			34	85
TM fasting		0.28	0.11		
TM non-fasting		0.30	0.11		
NIKBUT fasting		11.27	5.52		
NIKBUT non-fasting		10.33	4.01		
OSDI fasting		26	20		
OSDI non-fasting		20	18		

**Figure 1:**
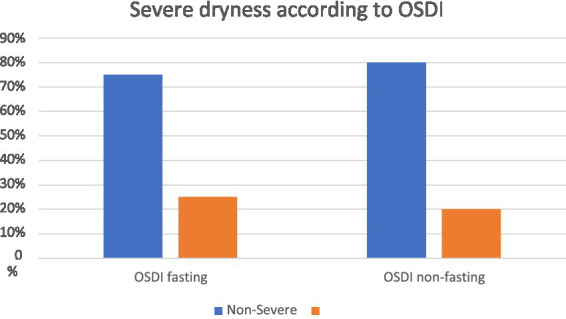
Comparison of severe dryness in The Ocular Surface Disease Index (OSDI) between fasting and non-fasting groups.

Upon comparing the dryness assessment variables during fasting and non-fasting periods, we found:

- Non-significant difference (*p* = 0.638) in OSDI, with a mean difference in OSDI score of 6.06 (95% CI 1.56 to 10.65).
- Non-significant difference (*p* = 0.484) in NIKBUT, with a mean difference of 0.95 (95% CI 0.90 to 2.86).
- Non-significant difference (*p* = 0.489) in TM, with a mean difference of −0.013 (95% CI 0.05 to 0.03) Upon comparing the percentage of participants who had severe dryness (i.e., OSDI ≥33) during fasting and non-fasting, we did not find a significant difference (*p* = 0.7889), with 10 (25%) participants had severe dryness during fasting compared to 8 (20%) during non-fasting (Figure 1).

**Figure 2:**
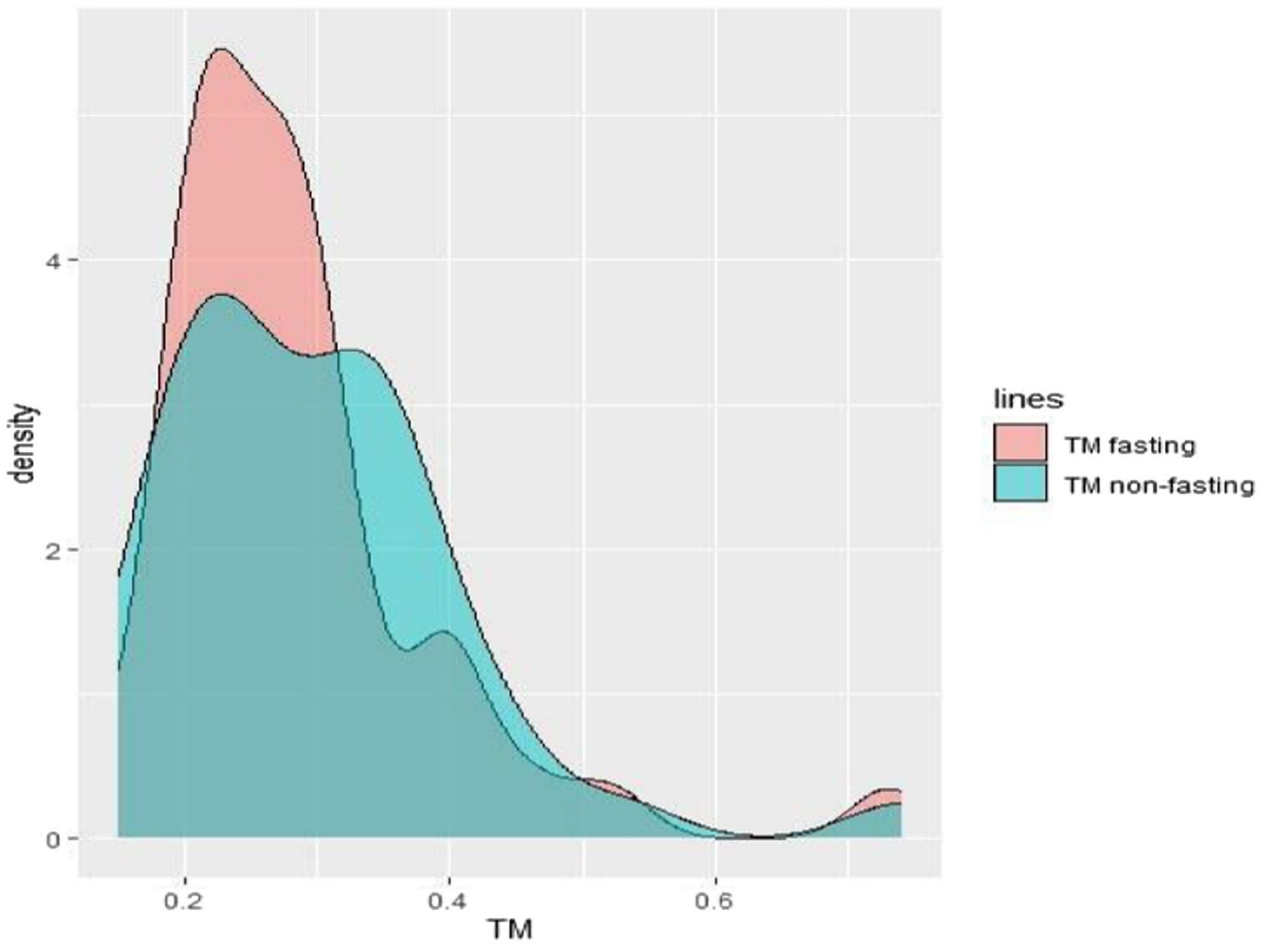
Density graph depicting the tear meniscus during both fasting and non-fasting states.

**Figure 3:**
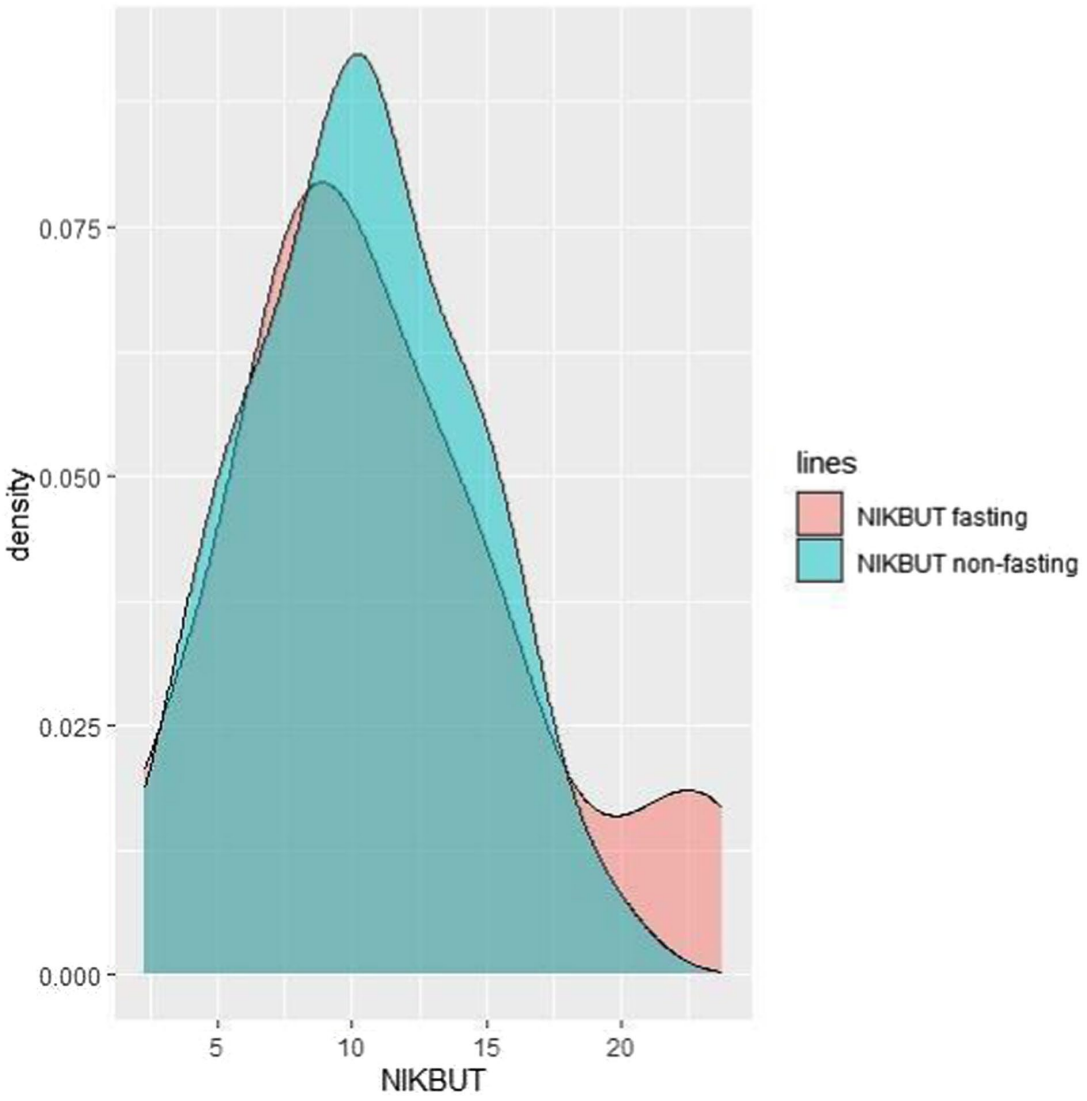
The density graph depicting the green curve (NIKBUT during non-fasting) consistently surpassing the red curve (NIKBUT during fasting) for 50% of the patients along the x-axis.

Regarding Tear Meniscus (TM) shown in Figure 2, the mean height observed during fasting periods is slightly lower than that recorded during non-fasting intervals. Furthermore, analysis of the TM graph reveals that 60% of the subjects (24 out of 40) exhibit higher TM values during non-fasting times compared to their values during fasting periods. Examining the density graph, it is evident that the green curve representing TM during non-fasting periods consistently surpasses the red curve denoting TM during fasting periods for 60% of the patients along the x-axis. For Non-Invasive Tear Break-Up Time (NIKBUT), as indicated in Figure 3 and Table 1, the mean NIKBUT value during fasting periods is 11.27, slightly elevated when contrasted with the mean NIKBUT value of 10.33 during non-fasting periods. A comparative analysis of individual patient data reveals that 50% of the subjects (20 out of 40) exhibit higher NIKBUT values during non-fasting times, signifying improved progression in NIKBUT values post-fasting.

We also compared the difference in dryness objective and subjective measures. We did not find a significant difference between men and women in terms of dryness during fasting. Regarding diabetes as a confounding factor, we did not find a significant difference between diabetes with ocular dryness parameters (see Table 2).

**Table 2:** Comparison between males and females in different dry eye related measures.

Gender		Mean	Std. Deviation	*p*-value
TM fasting *minus* non-fasting	Male	−0.35	0.16	0.349
	Female	−0.28	0.08	
NIKBUT fasting *minus* non-fasting	Male	0.21	5.16	0.426
	Female	1.50	6.77	
OSDI fasting *minus* non-fasting	Male	6.92	11.57	0.399
	Female	5.63	15.78	

## Discussion

In our study, 40 people were tested while they were fasting and again when they were not fasting. We used the Oculus Keratograph and a validated questionnaire to measure objective (TM and NIKBUT) and subjective (OSDI) dryness to see what effect dryness had. We did not find a significant difference in subjective dryness assessments on the OSDI scale, despite higher scores in fasting. Upon comparing the percentage of patients with severe dryness, we did not find a significant difference between fasting and non-fasting periods.

Moreover, we did not find a significant difference in the objective dryness measurement during fasting compared to non-fasting periods. Fasting may alter a variety of physiological parameters that by themselves influence the ocular system. We recommend that individuals with metabolic and other chronic diseases consult their physicians before fasting, even though fasting is safe for healthy individuals. Our main objective in this study was to understand the impact of prolonged fasting on eye dryness.

The demographic results of this study were similar to those of Armstrong et al. (6). In that study, 40 patients completed testing at both time points, with 13 men and 27 women. The mean age of those patients was 32, which is younger than the mean age of our patients. We did not find a difference in OSDI during fasting, which is consistent with Armstrong et al. (6). In contrast, Koktekir et al. (9), in a study conducted on 29 healthy men, found that OSDI was significantly increased during Ramadan with a *p*-value of 0.002.

In our cohort, the average NIKBUT values during the fasting times are a little bit higher than their NIKBUT values during the non-fasting times. Armstrong et al. (6) described a significant decrease in mean TBUT during the fasting times (with a *p*-value of 0.01). A study by Rabbanikhah et al. (13) found that basal tear secretion (BTS) and tear break time were both lower in the third week of Ramadan compared to the week before (*p* < 0.0001). This is similar to what Kerimoglu et al. (2) found, which is that basal tear secretion drops after 12 h of being dehydrated. In contrast, Kayikçioğlu et al. (8) examined 32 healthy male patients in winter and described no change in basal tear secretion (BTS) and tear break-up time (TBUT) in healthy individuals. In a study conducted on 94 healthy adults, Sedaghat et al. (14) also found no difference in TBUT during the fasting period. Regarding TM, the average in fasting time is lower than that in the non-fasting time but was statistically insignificant.

It is worth mentioning that the use of eyedrops a few hours before the test was significantly less in fasting patients, which might be a contributing factor to the results. This aligns with the findings of Kumar and Jivan (15), who reported a higher prevalence of non-adherence to prescribed eye care, such as lubricating eye drops, during Ramadan due to the belief held by 63.7% of patients that these drops break their fast. Moreover, Mahmoud et al. (16) described in a study on Nigerian fasting Muslims that 82.1% of patients prefer to minimize the use of eye drops in Ramadan to twice daily to avoid the use in fasting hours, and they also prefer to reschedule their appointments until Ramadan is over. Therefore, it is important to keep in mind that Ramadan could be an important cause for noncompliance with prescribed ophthalmic treatment, including lubricating eye drops, which may alter tear break-up time during this month.

The main limitation of our study is the small sample size included, despite being higher than the required sample size based on our priori calculation. We acknowledge that a larger cohort could enhance the generalizability of the results. However, the current study was designed as a preliminary investigation to explore the impact of prolonged fasting on dry eye disease, providing a foundation for future research with larger cohorts and a multicenter study for better recruitment. Longer fasting periods may as well yield additional insights for future studies to examine varying durations of fasting in larger populations.

Moreover, we assessed dryness at only two timepoints, during fasting and non-fasting. Dryness variability should be assessed at multiple time points during fasting and non-fasting to account for such variability.

## Conclusion

In conclusion, our comprehensive analysis of dryness assessment variables during both fasting and non-fasting periods revealed non-significant differences across key parameters.

This study’s outcomes suggest that the observed physiological changes in tear film parameters observed during Ramadan fasting do not result in significant differences in dryness-related variables. Despite variations in individual responses, the overall findings indicate a relatively consistent ocular response to fasting, emphasizing the need for further research to elucidate the complex interplay between fasting and ocular health.

## Data Availability

The raw data supporting the conclusions of this article will be made available by the authors, without undue reservation.
